# Diabetes prevalence in rural Indigenous Guatemala: A geographic-randomized cross-sectional analysis of risk

**DOI:** 10.1371/journal.pone.0200434

**Published:** 2018-08-09

**Authors:** Kent D. W. Bream, Amelia Breyre, Kristian Garcia, Erwin Calgua, Juan M. Chuc, Lynne Taylor

**Affiliations:** 1 Department of Family Medicine and Community Health, Raymond and Ruth Perelman School of Medicine, University of Pennsylvania, Philadelphia, Pennsylvania, United States of America; 2 Center for Global Health, Raymond and Ruth Perelman School of Medicine, University of Pennsylvania, Philadelphia, Pennsylvania, United States of America; 3 Center for Public Health Initiatives, University of Pennsylvania, Philadelphia, Pennsylvania, United States of America; 4 Harnwell College House, University of Pennsylvania, Philadelphia, Pennsylvania, United States of America; 5 Facultad de Ciencias Medicas, Universidad de San Carlos de Guatemala, Ciudad Guatemala, Guatemala; 6 Department of Emergency Medicine, Raymond and Ruth Perelman School of Medicine, University of Pennsylvania, Philadelphia, Pennsylvania, United States of America; 7 Health and Societies Program, Department of History and Sociology of Science, School of Arts and Sciences, University of Pennsylvania, Philadelphia, Pennsylvania, United States of America; 8 Hospitalito Atitlán, Canton Ch’utch’aj, Santiago Atitlán, Sololá, Guatemala; 9 Center of Excellence for Diversity in Health Education and Research, Raymond and Ruth Perelman School of Medicine, University of Pennsylvania, Philadelphia, Pennsylvania, United States of America; Johns Hopkins University Bloomberg School of Public Health, UNITED STATES

## Abstract

**Background:**

Developing countries and Indigenous populations are disproportionately affected by global trends in diabetes (T2DM), but inconsistent data are available to corroborate this pattern in Guatemala and indigenous communities in Central America. Historic estimates of T2DM, using a variety of sampling techniques and diagnostic methods, in Guatemala include a T2DM prevalence of: 4·2% (1970) and 8·4% (2003). Objectives of this geographically randomized, cross-sectional analysis of risk include: (1) use HbA1c to determine prevalence of T2DM and prediabetes in rural Indigenous community of Atitlán (2) identify risk factors for T2DM including age, BMI and gender.

**Methods:**

A spatially random sampling method was used to identify 400 subjects. Prevalence was compared using the confidence interval method, and logistic regression and linear regression were used to assess association between diabetes and risk factors.

**Findings:**

The overall prevalence of T2DM using HbA1c was 13·81% and prediabetes was also 13·81% in Atitlán, representing a tripling in diabetes from historic estimates and a large population with pre-diabetes. The probability of diabetes increased dramatically with increasing age, however no significant overall relationship existed with gender or BMI.

**Conclusions:**

Diabetes is a larger epidemic than previously expected and appears to be related to ageing rather than BMI. Our proposed explanations for these findings include: possible Indigenous unique genetic susceptibility to T2DM, shortcomings in BMI as a metric for adiposity in assessing risk, changes in lifestyle and diet, and an overall aging population. The conclusion of this study suggest that (1) T2DM in rural regions of Guatemala may be of epidemic proportion. With pre-diabetes, more than 25% of the population will be diabetic in the very near future; (2) Age is a significant risk factor in the Indigenous population but BMI is not. This suggests that in some populations diabetes may be a disease of ageing.

## Introduction

Even if the prevalence of obesity remains stable until 2030, it is anticipated that the number of people with diabetes globally will more than double since 2000 as a consequence of population aging and urbanization[1, 2]. Developing countries will be disproportionately affected by a global type 2 diabetes (T2DM) epidemic, but to what extent is limited by a paucity of prevalence data and an understanding of how risk factors may differ in different populations. This trend is of particular concern for Indigenous populations, who because of underlying genetic susceptibilities, have more severe metabolic consequences to a rapid change in nutrition and exercise than their non-Indigenous counterparts [3].

### T2DM in Guatemala

In Guatemala, historical data from 1970 estimates T2DM prevalence from 498 subjects in 48 sites to be 4·2% based on a glucose challenge test [4]. Over the past few decades, Guatemala has followed global trends toward increased sedentary lifestyles, erosion of traditional agricultural lifestyles [5]. In 2000, data compiled by the National Institute of Statistics of Guatemala estimated the regional mortality attributable to T2DM as 22% and cardiovascular disease as 38% [6]. In 2003, Pan American Health Organization-sponsored Central American Diabetes Initiative (CAMDI) estimated prevalence based on oral glucose tolerance tests (OGTT) in Villa Nueva, an urban predominantly mestizo population near Guatemala City, to be 8·4%. The data is similar to rates reported in Mexico City 8·7%, and greater than rates found in other cities in Latin America, such as La Paz, Bolvia (5·7%), Santiago Chile (6·5%), Bogotá, Colombia (7·4%); and Asunción, Paraguay (6·5%) [6–8].

In comparison to many Latin American countries, Guatemala has a proportionately large indigenous population. More than 60% of the population is indigenous, mainly concentrated in the western highlands of the country. Significant health disparities exist between the Indigenous and non-Indigenous populations for a variety of complex historical, economic, and social reasons [9, 10]. Prevalence data derived from predominantly urban populations may not accurately reflect the heterogeneity of national Guatemalan diversity. Therefore, this study reports a cross-sectional analysis of T2DM prevalence and risk factors in a rural population using novel geographic random sampling and HbA1c measurement.

## Materials and methods

### Study design and participants

This study was reviewed and approved (Protocol 817136) by the IRB of the University of Pennsylvania and was locally reviewed for ethical risk and approved by the Hospitalito Atitlán and the local Health Center in Guatemala. The sampling frame for this study consisted of adults living in the Maya Tz’utujil communities of San Pablo La Laguna, San Juan La Laguna, San Pedro La Laguna and the Maya Kaqchikel communities of San Antonio Palopó, Santa Catarina Palopó and San Lucas Tolimán (100,688 residents total) in the rural highland region of Atitlán, Guatemala. A representative sample of 400 participants–proportionally stratified by community based on population–was collected using a sampling technique based on a simple stratified random sampling design. Given that neither street addresses nor a list of households were available, a spatially random sampling methodology was developed in order to reduce opportunities for bias in the household selection process. Women who knew they were pregnant were excluded from the sample. Due to the exclusion of pregnant women, we did not consider gestational diabetes in the results.

Following this methodology, the research team obtained a gridded map of each community marking all households, businesses, major walkways and roads. For each map, a computer script generated a set of random coordinates within the community’s boundaries in proportion to the community’s population. In total 400 coordinates were generated and used by the research team to identify households from which participants would be recruited for this study.

Once the necessary random coordinates were generated and the points were plotted the following steps were taken by trained research staff accompanied by local clinical staff: (1) Approach the home or business nearest to each point. If reaching a certain point was impossible or posed a security risk, continue to the next point and generate a new random coordinate (2) Ask the first person 18 years or older who answers the door if they would like to participate in the study. If they do not wish to participate continue until a willing participant is found. A total of 400 participants provided verbal informed consent and were included in the study. Clinical risk factors for T2DM were obtained including: Body Mass Index (BMI), non-fasting capillary glucose and hemoglobin HbA1c were obtained from each participant. Definition of T2DM and prediabetes was used established HbA1c cutoffs (Table 1).

**Table 1:** Definition of DM and prediabetes based on American Diabetes Association [21].

	Pre-diabetes	Diabetes
Glycated hemoglobin (**HbA1c**)	5.7–6.4%	≥6.5%
Fasting plasma glucose (**FPG**). Fasting is defined as no caloric intake for at least 8h	5.6–6.9 mmol/L (100-125mg/dl) **Impaired fasting glucose (IFG**)	≥7.0mmol/L (≥126mg/dl)
2-hour plasma glucose in a 75g oral glucose tolerance test (**OGTT**)	7.8–11.0 mmol/L (140-199mg/dl) **Impaired glucose tolerance (IGT)**	≥11.1mmol/L (≥200mg/dl)
Patient with classic symptoms of hyperglycemia or hyperglycemic crisis Random plasma glucose (**PG**)		≥11.1mmol/L (≥200mg/dl)

BMI was calculated based on measurements of height in centimeters and weight in lbs. Standardized weights were measured using a battery powered digital scale. Height was measured using a standardized platform stadiometer. Blood samples were collected using venipuncture and transported in a cooler to a hospital based laboratory for immediate (same day) analysis. HbA1c was measured using the Quo-Lab® HbA1c test (EKF Diagnostics) which uses the boronate affinity methodology that is unaffected by Hb variants. HbA1c was recorded using the %DCCT. This method is standardised to the National Glycohemoglobin Standardization Program (NGSP) and is IFCC (International Federation of Clinical Chemistry) certified. The HbA1c was reported in %NGSP unit.

### Statistical analysis

We compared the prevalence of T2DM and prediabetes with the following three risks factor: (1) BMI (defined linearly and categorically as underweight, normal weight, overweight, obese), (2) gender and (3) age (defined linearly and categorically as <40yo, 40-64yo, 65+ yo) using the 95% CI interval method. We used multivariable logistic regression to assess the association between T2DM and each risks factors, while adjusting for other risks factors. Casewise deletion was used to handle missing data. The predicted odds and 95% CI of having T2DM, and the model’s c statistic were calculated. To describe the relationship between HbA1c levels and risk factors, unadjusted and adjusted multivariable linear regression was used. The beta coefficients and r-squares were computed. In this analysis, BMI and age are analyzed as continuous variables.

## Results

Four hundred subjects provided informed consent. One subject withdrew due to time constraints after consent. 371 subjects completed all measurements. Of the 28 that only provided partial data, 2 did not provide their age, 8 declined HbA1c measurement, and 22 were not able to participate in height or weight measure. The overall prevalence of T2DM was 13·81% and prediabetes was also 13·81% in Atitlán as diagnosed with HbA1c.(Tables 2 and 3)

**Table 2:** Studies of diabetes prevalence in Guatemala.

	West & Kalbfleish[4]	CAMDI[6]	Atitlán
Year	1970	2003	2015
Site(s) sampled in Guatemala	48 sites (rural/urban)	Villa Nueva, near Guatemala City (urban)	**Atitlán** **(rural)**
Sample selection technique	Random selection based on census data	Random selection based on census data	Random stratified geographic coordinates
Sample size	498 subjects	1397 enrolled 1047 (lab values)	400 enrolled 394 (lab values)
Subject ages	34+ years	20+ years	18+ years
Diagnostic test	Glucose Challenge	OGTT	HbA1c
DM Prevalence	4·2%	8·4%	13·81%

**Table 3:** Overall prevalence of DM and prediabetes.

Clinical status (HbA1c)	Atitlán (CI 95%) n = 391 [2015]	Guatemala City (CI 95%) (CAMDI n = 1,397)[8]* [2003]
Without diabetes (< 5.7)	72.38% (67.92–76.83)	64.6%
Prediabetes (5.7–6.4)	13.81% (10.38–17.25)	28.2%
Diabetes (≥6.5)	13.81% (10.38–17.25)	7.2%

*Diabetes and prediabetes determined by OGTT

This is compared to the 2003 CAMDI data, which used OGTT to report a 8·4% prevalence of T2DM in Guatemala City.(Table 3) Of the 400 participants, sampled the mean HbA1c was 5·85(sd = ± 1·78) age was 40·23(sd = ±15·29), and BMI was 26·73(sd = ±4·83). The average HbA1c for the group without diabetes was 5·14(sd = ±0·30), for the group with pre-diabetes was 5·91(sd = ±0·22) and the group with diabetes was 9·539sd = ± 2·49). The average age for the group without diabetes was 35·70(sd = ±13·49), the group with pre-diabetes was 50·28(sd = ±14·39) and the group with diabetes was 54·02(sd = ±12·44). The average BMI for the group without diabetes was 26·27(sd = ±4·66), the group with pre-diabetes was 27·92(sd = ±4·4) and for the group with diabetes was 28·16(sd = ±5·79) (Table 4).

**Table 4:** Overall mean of HbA1c, age and BMI in Atitlán.

					Overall mean ±STD: HbA1c, Age, BMI in non-diabetes, pre-diabetes, diabetes in Atitlán		
	N^a^	Min	Max	Overall Mean± sd	Non diabetes Mean± sd (n)	Pre-diabetes Mean± sd (n)	Diabetes Mean± sd (n)
HbA1c	391	4.3	14.9	5.85 ± 1.78	5.14±0.30 (283)	5.91±0.22 (54)	9.53 ± 2.49 (54)
Age	398	18	79	40.23±15.29	35.70±13.49 (283)	50.28±14.39 (54)	54.02±12.44 (54)
BMI	377	16.45	45.6	26.73±4.83	26.27±4.66 (270)	27.92±4.4 (51)	28.16±5.79 (50)

^a^400 persons enrolled in the study, 9 persons had missing HbA1C value. The above overall and subgroup sample sizes = persons with nonmissing values on the AIC and/or the age and bmi variable respectively.

In comparison to the CAMDI 2003 dataset, the Atitlán prevalence of diabetes and prediabetes is presented for each risk factor category (BMI, gender, age)(Table 5).

**Table 5:** Distribution of gender, age, BMI and respective DM/prediabetes prevalence in Atitlán and Guatemala City (CAMDI project).

Gender						
Distribution of Gender			Prevalence of prediabetes by gender (95% CI)		Prevalence of DM by gender (95% CI)	
	Atitlán	Guatemala City (CAMDI)[8]	Atitlán	Guatemala City (CAMDI)[8]	Atitlán	Guatemala City (CAMDI)[8]
M	30.9%	49.0%	17.07% (10.33–23.82)	25.4 (17.6–35.2)	12.20% (6.33–18.06)	7.8 (5.0–11.8)
F	69.1%	51.0%	12.31% (8.35–16.23)	30.8 (23.8–38.8)	14.56% (10.30–18.80)	6.8 (4.8–9.4)
**Age**						
Distribution of Age			Prevalence of prediabetes by age		Prevalence of DM by age	
18–39 yo	52.76%	68.2%	6.28% (2.95–9.61)	20.6% (15.4–26.8)	2.89% (0.59–5.20)	4.7% (2.7–8.1)
40–64 yo	37.4%	26.4%	19.31% (12.80–25.81)	40.3% (30.2–52.3)	23.45% (16.47–30.43)	14.5% (10.4–19.8)
65+ yo	9.8%	5.4%	33.33% (17.85–48.81)	27.6% (17.7–40.2)	35.90% (20.14–51.65)	17.5% (10.6–27.5)
**BMI**						
Distribution of BMI			Prevalence of prediabetes by BMI		Prevalence of DM by BMI	
Underweight (<18.5)	1.857%	1.5	-	-	14.29 (0.00–49.24)	-
Normal (18.5–24.9)	37.93%	33.1	9.29% (4.41–14.15)		10.00% (4.97–15.03)	3.1 (1.8–5.4)
Overweight (25–29.9)	40.05%	43.6	16.67% (10.63–22.70)		12.67 (7.28–18.05)	6.9 (4.0–11.6)
Obese (>30)	20.16%	21.8	17.57% (8.69–26.44)		21.62% (12.02–31.22)	11.5 (7.4–17.3)

A logistic regression compared gender, age categories (<40yo, 40-64yo, 65+yo), BMI categories (underweight, normal weight, overweight, obese) and the risk of T2DM (Table 6).

**Table 6:** Logistic regression of categorical gender, age, BMI and DM.

Variable	Odds Ratio (95% Confidence limits)	Significance (p-value) (c = 0.784)
Gender: Female vs Male*	0.85 (0.42–1.72)	0.651
Age: 40-64yo vs <40	**9.76** (3.93–24.24)	0.001
Age: 65+ vs <40	**18.47** (6.21–55.00)	0.001
BMI: underweight vs normal	1.02 (0.10–10.40)	0.988
BMI: overweight vs normal	1.34 (0.61–2.92)	0.458
BMI: obese vs normal	**2.38 (1.02–5.55)**	0.046

*Gender reference group = female

Age had a statistically significant overall relationship with T2DM. An individual over 65yo had an odds ratio of 18·47 (6·21–55·0) when compared to an individual below the age of 40(p < .001). Similarly, individuals 40-64yo versus individuals <40yo have a significantly higher predicted odds of T2DM (OR = 9·76, 95%CI = 3·93–24·24, p < .001). As BMI categories increased to overweight and obese the odds ratio of T2DM did as well, to 1·34 (0·61–2·92) and 2·38 (1·02–5·55) respectively. Only the comparison of obese to normal was considered statistically significant (p = 0.0452), however the overall effect of BMI to T2DM was not significant.

To examine the relationship between HbA1c and T2DM risk factors, we conducted unadjusted and adjusted linear regression. In contrast to Table 5, the analysis of HbA1c, age and BMI are treated as continuous linear variables rather than discrete categorical ones (Table 7).

**Table 7:** Unadjusted and adjusted relationship between HbA1c and DM Risk factors.

	Unadjusted (Linear bivariate Analysis)			Adjusted (Linear multivariate analysis)) (n = 371)^a^ r-square = 0.13		
Variable (n)	Beta coefficients	r-square	p-value	Beta coefficients	r-square	p-value
Gender (391)	-0.09*	0.001	0.6304	-0.131	0.001	0.4964
Age (391)	.0415	0.129	**< .0001**	0.043	0.129	**< .0001**
BMI (371)	0.026	0.005	0.1778	0.011	0.005	0.5660

*Gender reference group = female

^a^400 persons were enrolled in the study; 9 persons had a missing A1C value. The linear regression model is based on persons with no missing data on A1c, age, gender, and bmi (n = 371)

This alternative analysis using linear relationships for age and BMI were intended to be more reflective of the spectrum of biology naturally seen rather categorically described. The linear regression results indicates that age is significantly related to HbA1c (b = 0.04; p = 0.0001), while BMI and gender are not (p = 0.57, p = .50 respectively). Age accounts for 13% of the variance in HbA1c (r-square = 0.13), while BMI and gender account for less than 1% (r-square = 0.005, r-square = 0.0006 respectively).

## Discussion

### Age and DM

Age was the only variable in this study that demonstrated an overall significant relationship with T2DM and HbA1c. The mean age of study participants without diabetes (35·70) and with diabetes (54·02) differed by almost 20 years. The overall mean age CAMDI participants were approximately three years younger than our study participants–(CAMDI) 37·2 years old versus 40·23 years old. Age, but not BMI’s effect on the predicted HbA1c, is somewhat contrarian to conventional understanding of T2DM and perhaps warrants additional study on how T2DM should be perceived in different populations.

The existing body of literature that associates DM with aging proposes a variety of possible, likely multifactorial, mechanisms including: pancreatic beta- cell senescence [11, 12], epigenetic dysregulation of pancreatic islet cells [13], mitochondrial functional decline, increasing sarcopenia [14], increasing myosteatosis (skeletal muscle fat infiltration that occurs with advanced aging), increasing visceral fat[15] and reduced physical activity [16]. In a study of 35–84 year olds without diabetes, Hirose *et al* concluded that aging is an independent factor adversely affecting insulin concentrations, insulin resistance and beta-cell function [17]. Largely based on Hirose *et al*’s finding Okomato *et al* proposed a theoretical model of metabolic mechanisms that would explain how aging may affect insulin action independent of adiposity amount or even distribution. He suggests two hypotheses: (1) aging ameliorates insulin resistance by reducing hyperinsulinemia 2) aging causes beta-cell dysfunction/apoptosis which reduces insulin secretion [11].

In 1970, the average life expectancy was 52·05 years and currently is 72·02 years [18]. Although the increasing prevalence in T2DM in Guatemala is probably driven by the advanced aging of Guatemalans, it is also a population who are most at risk for the complications associated with T2DM including premature death and functional disability [19].

### BMI and DM

The traditionally strong relationship between BMI and T2DM was not evident in this study. Correlations were either weak or not statistically significant. The study protocol did not evaluate or exclude for type 1 DM. Known pregnancy was excluded as a vulnerable population. Although type 1 DM and gestational DM generally account for smaller portion of the population relative to T2DM, they do not necessarily demonstrate the same BMI patterns expected of T2DM [20, 21]. In addition, BMI is perhaps not the best metric for assessing obesity. The accuracy of BMI in diagnosing obesity is limited particularly for individuals in the intermediate range, in men and in the elderly [22]. Goh *et al* demonstrated that World Health Organization BMI definitions of obesity as a BMI >30, had a very low sensitivity in their local population of ethnic Chinese patients, and that sensitivity improved when establishing a BMI of 27 as local definition of obese [23]. Although redefining obese in the local Guatemalan population might be appropriate, the analysis of BMI as a linear value, instead of a categorical one, still did not yield any statistically significant relationship.

Shortcomings of BMI and the difficulty of characterizing adiposity between populations provide one possible explanation for the overall statistical un-relatedness of BMI and HbA1c. The “metabolically obese” phenotype, in which a greater tendency towards abdominal obesity and less muscle mass, results in an increased propensity for insulin resistance despite a relatively low prevalence of obesity [24]. Particularly in aging populations, where adiposity redistributions vary, BMI substantially underestimates the health-burden from excess adiposity [25]. Although some advocate the use of waist circumference as an alternative metric of adiposity, there may still be international population variability of this phenotype as demonstrated in a computer-based tomography study that concluded Asians have more visceral fat than Caucasians with the same waist circumference [26].

Another possible explanation for this non-relationship between BMI and T2DM might be that Indigenous Guatemalan physiology is fundamentally different to the physiology of individuals who form the majority of our T2DM knowledge base. Internationally, Indigenous people are a notoriously under-researched population who often face severe financial poverty and health disparities [5, 27–31]. The majority of the world’s Indigenous populations, in both developed and developing countries, have been experiencing a rapid increase in T2DM that outpaces their non-Indigenous counterparts [3, 32]. In addition the high cost of T2DM treatments like insulin, limited social support for dietary and lifestyle changes all create barriers that aggravate a T2DM epidemic [5].

Indigenous population social disparities may be further intensified by genetic susceptibilities to develop T2DM. In a Mexican mestizo population investigations of T2DM susceptibility loci for common European genetic variants identified 8 single nucleotide polymorphisms associated with T2DM including one genetic variation (CDKAL1) associated with the non-obese T2DM subgroup [33]. In a study specifically investigating the inherited component of T2DM in Indigenous Mexico, several genetic polymorphisms were identified for their association for T2DM in Maya population [34]. No similar studies exist for Guatemalan populations.

### Gender and DM

There was no significant difference in T2DM and HbA1c between males and females. This is consistent with data predominantly from the US and UK where there is not thought to have an overall gender bias [35]. There is a notable female skew in this study where 69·1% of participants were female. This might reflect a selection bias of the novel randomly generated geographic sampling method in which participants included in the study are those at home during the day.

### Defining diabetes and prediabetes

One of the major challenges in contextualizing and interpreting prevalence of T2DM and prediabetes is the variety of definitions and methods used (Table 1, Table 2). The West & Kalbfleish 1970 the assessment of T2DM in Guatemala defined diabetes using the outdated oral glucose load test, in which an oral glucose load test, all subjects received an oral glucose load of 1gm/kg of body weight and venous blood was drawn at two hours [4]. The CAMDI studies used the oral glucose tolerance test (OGTT), in which after 12 hours of fasting 75g of glucose is given to a subject. After two hours the blood plasma levels are measured [6].

The adoption of HbA1c tests to diagnose diabetes has been slow and somewhat controversial, but research benefits include being logistically simpler and faster value to acquire from study participants and that its standard would facilitate comparison of research [36–38]. Current HbA1c thresholds for diagnostic criteria in T2DM and prediabetes (T2DM ≥ 6·5, pre-diabetes ≥5·7) are established because those levels are associated with certain benchmarks of microvascular and macrovascular disease. However, it is recognized that risk is continuous extending below the lower limit of the range and becoming disproportionately greater at higher ends of the range [21, 37, 39, 40]. This makes the mean group with diabetes HbA1c of 9·53 in this study, particularly alarming. Those who have diabetes in this Guatemalan population are very poorly controlled and thus profoundly at risk for the microvascular and macrovascular complications of T2DM.

The 13·81% prevalence of pre-diabetes identified in this study using HbA1c definition of ≥5.7, is a stark contrast to the 28.2% of individuals with OGTT in the CAMDI study. This underscores the aforementioned difficulty in comparing data in studies using different but valid methods of T2DM assessments. Prediabetes is an important epidemiological assessment of understanding a T2DM epidemic. Prediabetes places individuals at higher risk of developing T2DM and its complications. The annual conversion of prediabetes to T2DM is 5% [40, 41]. Moreover, prediabetes, independently of T2DM, is an important entity to identify because subsequent intervention of prediabetes can prevent progression to diabetes. Not only is prediabetes of important prognostic value in understanding diabetes risk but it also represents an ideal target population for diabetes prevention.

### Strengths and limitations

This cross-sectional study used a geographically randomized sample to assess the prevalence of diabetes in a rural predominantly ethnic minority population in Guatemala that revealed significant increases in diabetes prevalence from previous studies. This correlates to the reported increase in diabetes related complications being experienced in Guatemala currently. A unique strength of this study is the use of HbA1c to screen for diabetes in concordance with current international guidelines. Despite the important public health strengths of the paper, some limitations should be considered in interpreting the results. First, HbA1c may be impacted by hemoglobin genetic variants as well as anemia. While this may impact total prevalence, genetic variation should not vary with age but instead with at risk population. In addition, the HbA1c analysis method used is not impacted by hemoglobin variations. Finally, while anemia is prevalent in the population, this has been primarily observed in the pediatric and pregnant population which was not included in the current population. This would be an area of more in depth study to understand the global epidemic of diabetes. Second, the study population was dominated by the Tz’utujil and Kaqchikel Maya and may not be generalizable across all Hispanic populations. Expanding previous analyses from the urban areas to the rural areas expands, however, the understanding of the global diabetes epidemic rather than limits it. Third, the lack of correlation with BMI is concerning given the historic findings regarding obesity and diabetes. This finding is surprising and warrants more careful study to assess the potential causative factors and confounders of diabetes. This study was designed to assess the prevalence of DM in an underserved population and indicates that additional studies are needed. While each of these limitations are important, we believe the larger public health crisis that is suggested by the growing prevalence of diabetes globally underlines the importance of our findings. Access to effective and simplified screening tools will assist in patients identifying their status regarding diabetes to implement the medical and behavioral interventions to prevent morbidity and mortality.

## Conclusions

Historical T2DM prevalence data in Guatemala is limited to a 1970 multi-site study yielding a 4·2% prevalence based on a glucose load test[4], a 2003 urban mestizo site study yielding 8·4% based on OGTT [6–8]. The results of this study report a 13·81% prevalence of DM in an Indigenous, rural community of Atitlán. There are numerous considerations in comparing these various data points–different populations, different sampling methods, different measuring tools (Table 2), and different definitions of diabetes (Table 1). The overall qualitative trend demonstrates a concerning increase of DM in Guatemala associated with increasing age and it may represent a health disparity in Indigenous populations.

The reasons for this trend is likely multifactorial—increased sedentary lifestyles, change in diet, an aging population and perhaps an underlying genetic difference. There is, however, surprisingly no correlation with BMI. Further investigation of specific ways Guatemalan lifestyles have changed, adipose distribution, correlation with anemia, and genetic risk factors is required to understand environmental and genetic influences. While an early comparative study in 1962 looked at Guatemala and the US and diabetes [42], the findings of this study suggest further understanding is still needed more than 50 years later.
